# REFRAMING AGING: UNIVERSITIES AS AN ENGINE FOR EVIDENCE-BASED INTERGENERATIONAL APPROACHES

**DOI:** 10.1093/geroni/igz038.2332

**Published:** 2019-11-08

**Authors:** Karl Pillemer

**Affiliations:** Cornell University, Ithaca, New York, United States

## Abstract

Societal changes are decreasing opportunities for youth to engage with older adults. Geographical mobility, the digital divide, and the growth of age-segregated communities for older people increase age segregation. The lack of interaction can lead to negative attitudes and stereotypes among young and older people. A solution is increasing meaningful contact between youth and older adults. This presentation proposes that higher education can play a unique role in reframing aging through intergenerational programs. Colleges and universities can integrate innovative bodies of research and practice, foster more rigorous research designs to study the effectiveness of intergenerational programs; and discover new ways to provide youth with needed skills and knowledge on how to interact with older adults. The national Cooperative Extension System is presented as a model for how evidence-based practice in intergenerational programs can be translated to communities, with a focus on collaborative program design, evaluation, and broader uptake.

